# Cancer RNA-Seq Nexus: a database of phenotype-specific transcriptome profiling in cancer cells

**DOI:** 10.1093/nar/gkv1282

**Published:** 2015-11-23

**Authors:** Jian-Rong Li, Chuan-Hu Sun, Wenyuan Li, Rou-Fang Chao, Chieh-Chen Huang, Xianghong Jasmine Zhou, Chun-Chi Liu

**Affiliations:** 1 Institute of Genomics and Bioinformatics, National Chung Hsing University, Taichung 402, Taiwan; 2 PhD Program in Medical Biotechnology National Chung Hsing University, Taichung 402, Taiwan; 3 Molecular and Computational Biology, University of Southern California, Los Angeles, CA 90089, USA; 4 Department of Life Sciences, National Chung Hsing University, Taichung 402, Taiwan

## Abstract

The genome-wide transcriptome profiling of cancerous and normal tissue samples can provide insights into the molecular mechanisms of cancer initiation and progression. RNA Sequencing (RNA-Seq) is a revolutionary tool that has been used extensively in cancer research. However, no existing RNA-Seq database provides all of the following features: (i) large-scale and comprehensive data archives and analyses, including coding-transcript profiling, long non-coding RNA (lncRNA) profiling and coexpression networks; (ii) phenotype-oriented data organization and searching and (iii) the visualization of expression profiles, differential expression and regulatory networks. We have constructed the *first* public database that meets these criteria, the Cancer RNA-Seq Nexus (CRN, http://syslab4.nchu.edu.tw/CRN). CRN has a user-friendly web interface designed to facilitate cancer research and personalized medicine. It is an open resource for intuitive data exploration, providing coding-transcript/lncRNA expression profiles to support researchers generating new hypotheses in cancer research and personalized medicine.

## INTRODUCTION

Over the past two decades, gene expression data generated by microarray technology has been widely used to study the causes and therapies of cancers. Research on the human transcriptome has produced a large amount of expression data at the gene level. Many gene-expression databases have been developed for cancer research, such as Oncomine (public version) (1), NextBio (2) and GCOD (3). However, a given gene is often spliced into multiple transcript isoforms, which in turn are translated into different proteins. More than 90% of human genes undergo alternative splicing (4). Therefore, the gene-level expression data generated by microarray platforms are insufficient to understand the involvement of specific proteins in cancer. RNA-Seq (5) is a revolutionary tool that can be used to study alternative splicing and to quantify gene/isoform expression levels across a genome. RNA-Seq has been widely used in many cancer studies.

Several major data portals contain cancer RNA-Seq data, such as the NCBI Gene Expression Omnibus (GEO) (6) and the Sequence Read Archive (SRA) (7). However, these portals mainly serve as raw data archives. They do not provide the full utility of RNA-Seq data for biologists, because highly developed bioinformatics skills are required to set up and manipulate data pipelines, tune parameters and control quality during data processing, analysis and visualization. The Cancer Genome Atlas (TCGA) (http://cancergenome.nih.gov/) database stores genomics data (including RNA-Seq data) for a variety of cancer types, but it only contains data generated by TCGA consortium. The RNA-Seq Atlas (8) attempts to provide easy access to RNA-Seq expression profiles. However, it only has one RNA-Seq data set and 11 samples. Recently, we have constructed a database for isoform–isoform interactions using 19 RNA-Seq data sets (9), and performed high-resolution functional annotation of the human transcriptome using 29 RNA-Seq data sets (10). Nevertheless, these studies have not utilized long non-coding RNA (lncRNA) expression profiles, which we believe is complementary to the coding-transcript expression data in cancer research.

**Figure 1.**
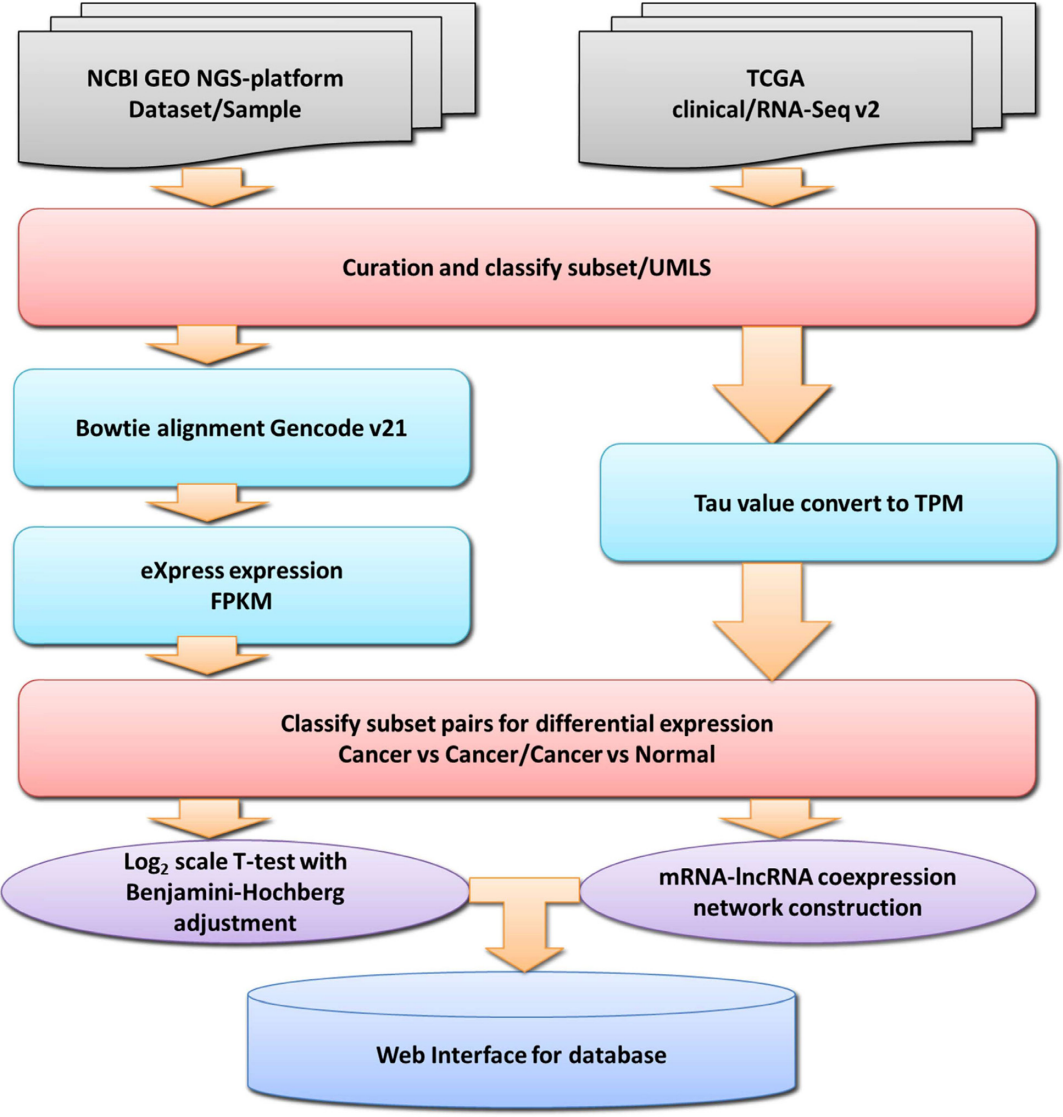
Framework for constructing the CRN database. Cancer RNA-Seq data sets were collected from NCBI GEO, SRA and TCGA, and then all samples were classified into the phenotype-specific subsets. For the GEO data sets, Bowtie2 and eXpress software were used to calculate isoform expressions using GENCODE v21 as a reference. For the TCGA data sets, we converted the expression values (tau values) of the TCGA Level 3 RNA-Seq version 2 data sets to TPM (transcripts per million). To identify phenotype-specific differentially expressed protein-coding transcripts and lncRNAs in each data set, we performed log_2_ scale *t*-tests with Benjamini–Hochberg adjustment between each pair of subsets with no overlapping samples and from the same data set. For each subset pair, we selected coding transcripts and lncRNAs with high expression variance, then calculated the correlations of expression profiles between selected coding transcripts and lncRNAs to construct an mRNA–lncRNA coexpression network.

This paper presents the Cancer RNA-Seq Nexus (CRN), the first public database providing phenotype-specific coding-transcript/lncRNA expression profiles and mRNA–lncRNA coexpression networks in cancer cells. CRN includes cancer RNA-Seq data sets in the TCGA, SRA and GEO databases. Figure 1 shows the framework that we used to construct the database. It resulted in 54 human cancer RNA-Seq data sets, including 326 phenotype-specific subsets and 11 030 samples. Each subset is a group of RNA-Seq samples associated with a specific phenotype or genotype, e.g. breast cancer stage II, ER+ breast cancer or Her2+ breast cancer. This specificity facilitates research into personalized medicine. CRN provides a user-friendly interface to efficiently organize and visualize coding-transcript/lncRNA expression profiles. It also permits the visualization and analysis of mRNA–lncRNA coexpression networks for any pair of phenotype-specific subsets. CRN is freely available at http://syslab4.nchu.edu.tw/CRN.

## MATERIALS AND METHODS

### RNA-seq data sets

We collected a total of 54 human cancer data sets and 11 030 samples from major cancer RNA-Seq data portals, including the Gene Expression Omnibus (GEO) (6), Sequence Read Archive (SRA) (7) and the Cancer Genome Atlas (TCGA http://cancergenome.nih.gov/). The final database consists of 28 TCGA data sets and 26 GEO/SRA data sets.

#### GEO/SRA data sets

We obtained annotation data for the RNA-Seq data sets from GEO, and downloaded RNA-Seq reads from SRA. Each data set consists of several subsets, where each subset is a group of RNA-Seq samples associated with a specific phenotype trait or cancer condition. For example, the subsets can be identified with a specific stage of cancer, a disease state, a cell line, a type of tissue or a genotype. We manually created these subsets from the raw data and annotations, assigning samples based on their textual descriptions. The GEO/SRA data yielded a total of 142 subsets and 1337 samples. To obtain expression profiles for both coding transcripts and lncRNAs, we downloaded the entire human transcriptome with Ensembl ID, including 93 139 protein-coding and 26 414 lncRNA transcripts, from GENCODE (Release 21, GRCh38) (11) as a reference. Bowtie (12), version 1.1.1, was used to build the reference index and align the RNA-Seq reads to the reference transcriptome. To ensure enough depth of sequencing coverage, we only selected samples with at least 1 million reads and 15% of reads mapped to the reference transcriptome (13). After this step, isoform abundances in each sample were quantified using eXpress (14), version 1.5.1, with the expression unit ‘fragments per kilobase of transcript per million mapped reads’ (FPKM) (15).

#### TCGA data sets

We collected all Level 3 RNA-Seq version 2 data sets and clinical information from the TCGA portal (http://cancergenome.nih.gov/). We defined subsets for all patient samples based on TCGA clinical information, e.g. primary tumor, metastatic, recurrent tumor, adjacent normal tissue or the pathologic tumor stage. Since the number of adjacent normal samples for each tumor stage is small, we pull all the adjacent normal samples from various tumor stages into a normal subset for each TCGA data set. This analysis resulted in a total of 239 subsets and 10,270 samples. The TCGA expression data contain 67 119 transcripts with UCSC KnownGene IDs that are given gene symbol assignments according to the UCSC KnownGene annotation (16). Since GENCODE provides annotation of protein-coding transcripts and lncRNAs, we classified these KnownGene IDs into 63 775 protein-coding transcripts and 3344 lncRNAs using the mapping of GENCODE and KnownGene IDs. We multiplied the expression values (tau values, calculated by RSEM) (17) of the TCGA Level 3 RNA-Seq version 2 data sets by 10^6^ to obtain transcripts per million (TPM).

### Phenotype-specific differentially expressed transcripts (DETs)

To identify phenotype-specific differentially expressed (DE) protein-coding transcripts and DE lncRNAs, within each data set we selected all subsets with at least three samples, then converted the expression value to log_2_ scale with added pseudo-counts and performed a *t*-test between every pair of subsets with no samples in common. We used Benjamini–Hochberg adjustment (18,19) to calculate the adjusted *P*-value. There were 601 subset pairs with differentially expressed transcripts (adjusted *P*-value < 0.01): 444 ‘cancer versus cancer’ and 157 ‘cancer versus normal’ subset pairs.

### Construction of mRNA–lncRNA coexpression networks

To investigate the phenotype-specific regulation of mRNAs and lncRNAs, which may reveal the potential biological functions of lncRNAs, we constructed mRNA–lncRNA coexpression networks. For a given subset pair, we selected coding transcripts and lncRNAs with high expression variance (standard deviation > 0.3 and (standard deviation / mean) > 0.5), then calculated correlations between every pair of selected mRNA and lncRNA expression profiles. The samples of the subset pair were used for the correlation analysis. To assess the significance of these connections, we transferred Pearson's correlation coefficient *r* to a *P*-value using the following method: given a sample size *n*, we calculated the *t*-value (20):

\begin{equation*} t = \frac{r}{{\sqrt {\frac{{1 - r^2 }}{{n - 2}}} }} \end{equation*}

The *P*-value was calculated using this *t*-value for the *t*-distribution with *n*−2 degrees of freedom. The significant correlations between protein-coding transcripts and lncRNAs (*P*-value < 1E-6) were collected for network construction. In the web interface, Cytoscape (21) was used to demonstrate the mRNA–lncRNA coexpression networks. In the web interface, users can input a gene symbol and an lncRNA name, then select a correlation threshold.

## WEB INTERFACES

**Figure 2.**
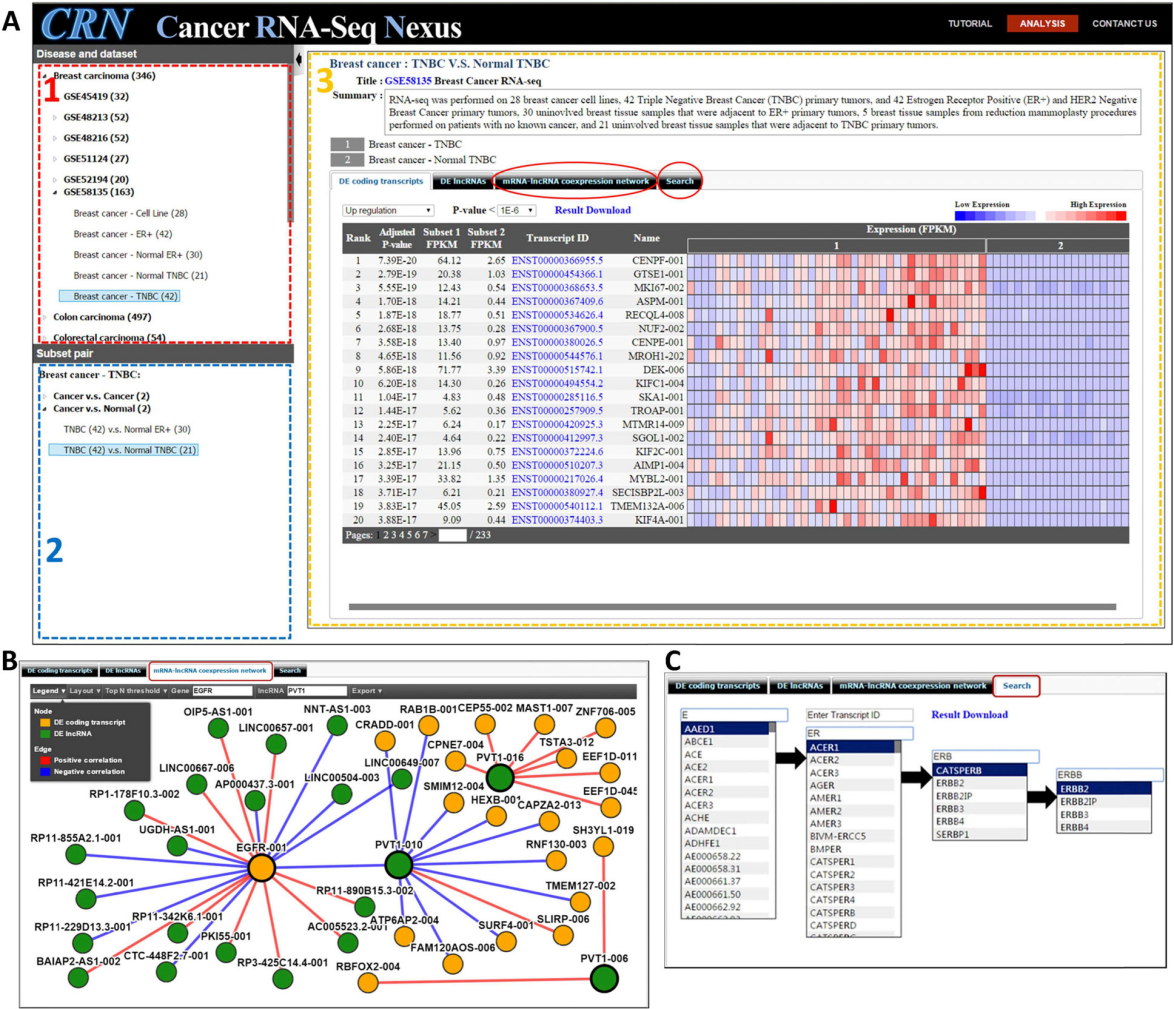
Screenshots of the web interface of CRN. **(A)** The CRN web interface provides three major panels: (1) Disease-dataset panel (upper left). The hierarchical menu illustrates which subsets are associated with which cancer types. A subset is a group of RNA-seq samples associated with a specific phenotype or genotype, e.g. breast cancer stage II, *ER*+ breast cancer or *Her2*+ breast cancer. (2) Subset-pair panel (bottom left). There are two types of subset pairs: ‘Cancer versus Cancer’ and ‘Cancer versus Normal’. When users select a subset in the first panel, CRN shows all associated subset pairs in this panel. (3) Profile panel (right). After clicking a subset pair in the second panel, CRN displays a detailed description of the data set and subsets. In this panel, there are four analysis tabs: (i) DE coding transcripts visualizes the expression profiles of differentially expressed (DE) protein-coding transcripts. (ii) DE lncRNAs visualizes the expression profiles of DE lncRNAs. (iii) mRNA–lncRNA coexpression network The user can search for a specific gene and lncRNA in this tab, then visualize the most significant negative and positive correlations between coding transcripts and lncRNAs as a network **(B)**. (iv) Search Users can search for gene/transcript names and transcript IDs. This panel provides an auto-complete function which displays partially matched terms as the user types, filtering the list to provide better and better matches **(C)**. After selecting a gene or transcript, the panel shows the expression profiles.

We developed a user-friendly web interface to present the CRN database, which integrates large-scale RNA-Seq data sets and cancer phenotype/genotype information. As shown in Figure 2A, the CRN database displays a hierarchical menu in the disease-dataset panel (upper left) to help users search and browse the different phenotype-specific subsets. When users select a cancer type or a cancer subset, the associated subset pairs are displayed in the bottom-left panel. After a user selects a subset pair, the web server shows a complete description of the data set/subsets, and permits analysis of the data through four tabs in the right panel:

- **DE coding transcripts**. Given a subset pair, CRN visualizes the expression profiles of all differentially expressed (DE) protein-coding transcripts, sorted by the significance level (adjusted *P*-value) between two subsets. Users can filter DE transcripts based on their adjusted *P*-value threshold or up/down regulation. The interface displays the rank, the adjusted *P*-value, the average expression values, the transcript ID and the gene symbol for each transcript. The transcript ID represents Ensembl transcript ID for GEO/SRA data sets and it represents UCSC KnownGene ID for TCGA data sets.
- **DE lncRNAs**. Given a subset pair, CRN visualizes the expression profiles of DE lncRNAs sorted by the significance level between two subsets. Users can filter DE lncRNAs based on an adjusted *P*-value threshold or up/down regulation. CRN displays the rank, the adjusted *P*-value, the expression values of two subsets, the lncRNA ID and the lncRNA name.
- **mRNA–lncRNA coexpression network**. Given a subset pair, CRN visualizes an mRNA–lncRNA co-expression network, displaying coding transcripts and lncRNAs with significant correlations between coding transcripts and lncRNAs (illustrated in Figure 2B). Users can input a gene symbol and an lncRNA name, then select a correlation threshold. They can also choose how many of the most significant connections to show (*N* = 10, 15, 20 or 25) for the given gene or the given lncRNA.
- **Search for coding transcripts and lncRNAs**. Given a gene symbol, CRN provides a search function on the expression profiles of all transcripts associated with the given gene, allowing users to investigate the differential expressions of its various isoforms. An auto-complete function provides suggestions for gene or transcript symbols as the user types, quickly searching and displaying partially matched terms. The auto-complete function not only helps users search efficiently, but also make a quick filtering (illustrated in Figure 2C).

## EXAMPLE APPLICATIONS

To demonstrate the biological applications of CRN, we used the genes *TP63*, *CCAT1* and *MYC/PVT1* as examples to show the functionality of differentially expressed coding transcripts, differentially expressed lncRNAs and the mRNA–lncRNA coexpression network.

### Differentially expressed coding transcripts

**Figure 3.**
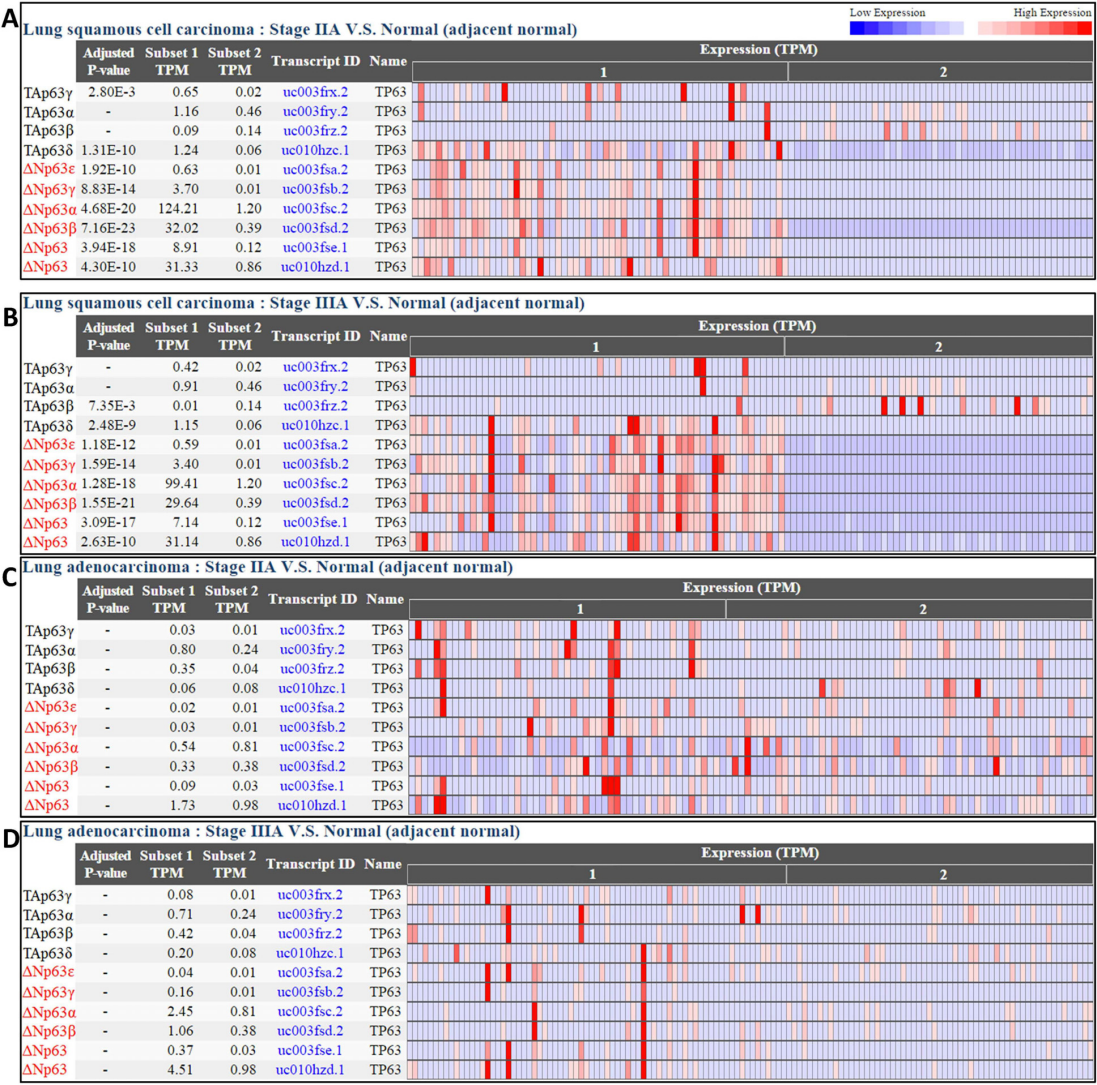
The differential expressions of two *TP63* major isoform groups ‘*TAp63* and *ΔNp63*’ in lung squamous cell carcinoma **(A, B)** and lung adenocarcinoma **(C, D)**. Based on the adjusted *P*-values calculated by CRN, *ΔNp63* isoforms are significantly overexpressed with respect to normal tissue in lung squamous cell carcinoma, but not in lung adenocarcinoma. In contrast, *TAp63* has low expression in all four subset pairs. In the adjusted *P*-value column, the absent values indicate insignificance (adjusted *P*-value > 0.01) in differential expression analysis.

To demonstrate the biological function of DE protein-coding transcripts in CRN, we use the *TP63* gene as an example. *TP63* has multiple transcript isoforms as a result of alternative promoter usage and alternative splicing. Its transcript isoforms fall into two groups: (i) the upstream promoter of the gene generates *TAp63* isoforms containing the N-terminal transactivation (TA) domain, and (ii) an alternative internal promoter generates *ΔNp63* isoforms lacking the TA domain (22). In each group, additional variants (e.g. *α*, *β* and *γ*) with various C-terminal tails are generated through alternative splicing at the 3′ end. It has been suggested that *ΔNp63* isoforms are highly specific for squamous cells: they are overexpressed in squamous cell carcinoma (SCC) (23–25), while *TAp63* expression is either lost or extremely low in squamous cells and SCC (25,26). Therefore, *ΔNp63* may be used to precisely distinguish between lung adenocarcinoma and lung SCC, allowing for novel targeted therapies (27,28). As shown in Figure 3, the database confirms that all *ΔNp63* isoforms are significantly overexpressed with respect to normal tissue in lung SCC, while the *TAp63* isoforms are not. On the other hand, *ΔNp63* isoforms are not differentially expressed between cancer and normal subsets in lung adenocarcinoma.

Table 1 shows the average transcript expressions of the *TAp63α* and *ΔNp63α* isoforms (those with the longest 3′-terminus) in lung SCC and lung adenocarcinoma. The expression of *ΔNp63α* is extremely high (Average TPM = 109.61) and significantly overexpressed (adjusted *P*-value = 8.12E-68) in lung SCC, compared with normal samples (Average TPM = 1.205). The expression of *ΔNp63α* is very low (Average TPM = 1.229 in cancer, 0.807 in normal) and has no significant differential expression (adjusted *P*-value = 3.00E-1) in lung adenocarcinoma. The expression of *TAp63α* is extremely low in both lung SCC and lung adenocarcinoma (Average TPM = 0.239–0.833), consistent with the previous reports (23–25).

**Table 1.** The transcript expressions of *TAp63α* and *ΔNp63α* in lung squamous cell carcinoma and lung adenocarcinoma

Subset pair	Isoform ID	Isoform name	Average expression in cancer	Average expression in normal	Adjusted *P*-value
Lung squamous cell carcinoma versus adjacent normal	uc003fry.2	TAp63α	0.833	0.456	1.22E-01
Lung squamous cell carcinoma versus adjacent normal	uc003fsc.2	ΔNp63α	109.610	1.205	8.12E-68
Lung adenocarcinoma versus adjacent normal	uc003fry.2	TAp63α	0.646	0.239	1.35E-01
Lung adenocarcinoma versus adjacent normal	uc003fsc.2	ΔNp63α	1.229	0.807	3.00E-01

### Differentially expressed lncRNAs

The Colon Cancer Associated Transcript-1 (*CCAT1*) lncRNA is significantly up-regulated in colon and colorectal cancer compared with the normal human tissues (29,30). Recently, *CCAT1* has also been found to be highly up-regulated in gastric carcinoma, compared with adjacent normal gastric tissues (31,32). Our data analysis confirms these findings. Specifically, there are seven colon adenocarcinoma subset pairs of the type ‘Cancer versus Normal’ in CRN. In all these pairs, *CCAT1* is significantly overexpressed (adjusted *P*-values 9.48E-10–1.35E-50) in cancer subsets (average TPM = 4.53–9.89) with respect to normal subsets (average TPM = 0.318) (Table 2A). Additionally, there are eight stomach adenocarcinoma subset pairs categorized as ‘Cancer versus Normal’ in CRN, and *CCAT1* is significantly overexpressed (adjusted *P*-values 5.02E-3–4.77E-8) in seven cancer subsets (average TPM = 4.43–10.17) with respect to normal subsets (average TPM = 1.575) (Table 2B). Interestingly, the expression values of *CCAT1* are extremely similar between colon adenocarcinoma and stomach adenocarcinoma, consistent with the previous reports.

**Table 2.** The lncRNA expression of *CCAT1* in colon adenocarcinoma and stomach adenocarcinoma

	Average expression in cancer	Average expression in normal	Adjusted *P*-value
**A. Colon adenocarcinoma subset pair**			
Colon adenocarcinoma—Stage I versus Normal (adjacent normal)	8.584	0.318	1.67E-34
Colon adenocarcinoma—Stage II versus Normal (adjacent normal)	9.887	0.318	7.99E-13
Colon adenocarcinoma—Stage IIA versus Normal (adjacent normal)	6.926	0.318	1.35E-50
Colon adenocarcinoma—Stage III versus Normal (adjacent normal)	4.527	0.318	9.48E-10
Colon adenocarcinoma—Stage IIIB versus Normal (adjacent normal)	8.182	0.318	8.00E-23
Colon adenocarcinoma—Stage IIIC versus Normal (adjacent normal)	9.078	0.318	4.12E-13
Colon adenocarcinoma—Stage IV versus Normal (adjacent normal)	7.583	0.318	4.17E-29
**B. Stomach adenocarcinoma Subset Pair**			
Stomach adenocarcinoma—Stage IA versus Normal (adjacent normal)	4.986	1.575	9.48E-04
Stomach adenocarcinoma—Stage IB versus Normal (adjacent normal)	5.815	1.575	5.90E-04
Stomach adenocarcinoma—Stage II versus Normal (adjacent normal)	4.430	1.575	5.02E-03
Stomach adenocarcinoma—Stage IIA versus Normal (adjacent normal)	5.615	1.575	1.79E-02
Stomach adenocarcinoma—Stage IIB versus Normal (adjacent normal)	5.809	1.575	7.91E-06
Stomach adenocarcinoma—Stage IIIA versus Normal (adjacent normal)	7.738	1.575	4.77E-08
Stomach adenocarcinoma—Stage IIIB versus Normal (adjacent normal)	7.857	1.575	2.56E-05
Stomach adenocarcinoma—Stage IV versus Normal (adjacent normal)	10.166	1.575	3.50E-06

### mRNA–lncRNA coexpression network

**Figure 4.**
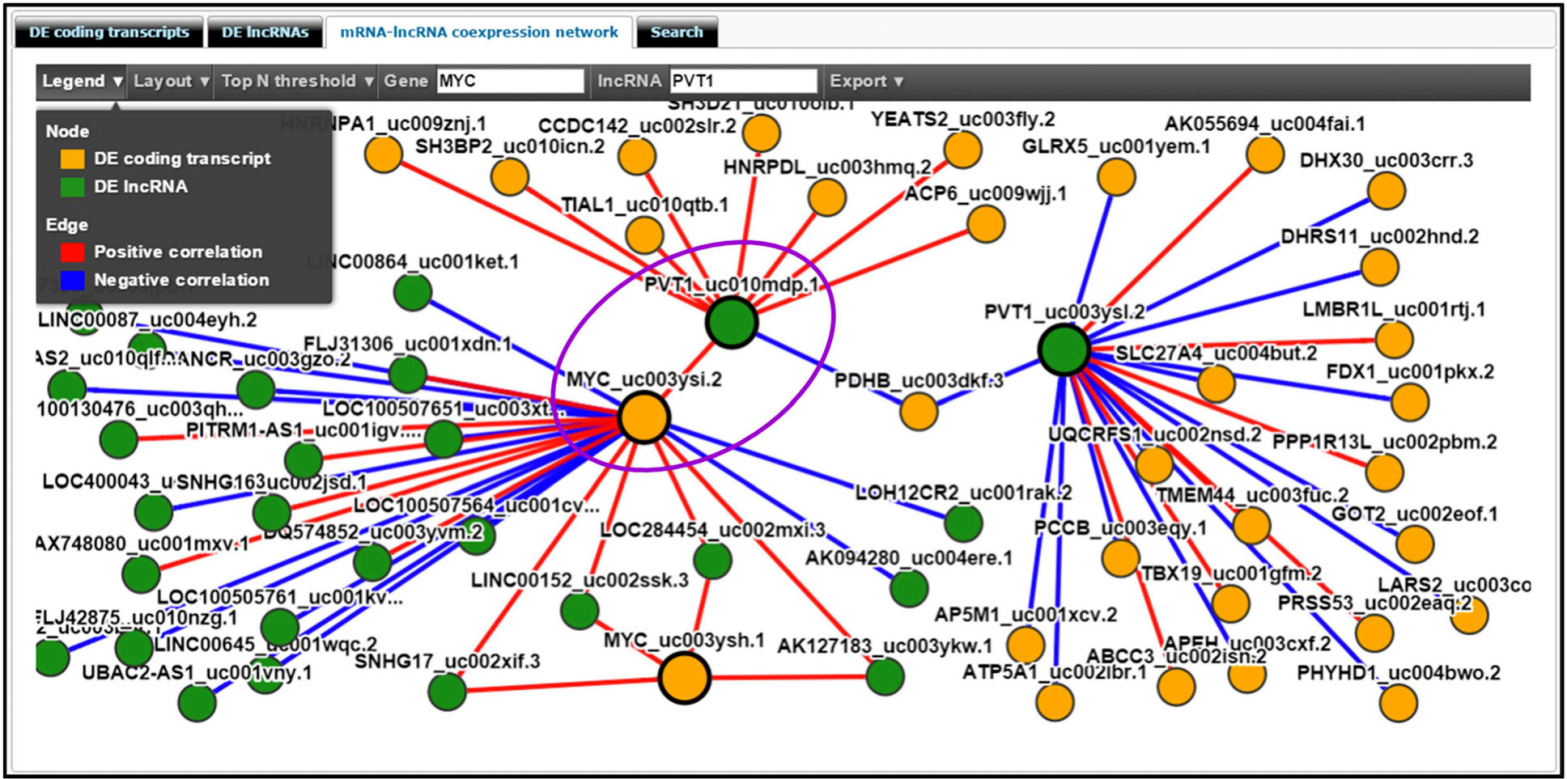
The coexpression network of the gene *MYC* and lncRNA *PVT1* in the subset pair of kidney renal clear cell carcinoma: Stage I V.S. Adjacent normal. There is a significant positive correlation between *MYC* isoform *uc003ysi.2* and *PVT1* isoform *uc010mdp.1*. *MYC* has two isoforms, *uc003ysh.1* and *uc003ysi.2*, and both of them are significantly overexpressed in the subset of kidney renal clear cell carcinoma: Stage I versus adjacent normal (adjusted *P*-values 1.25E-14 and 4.56E-17, respectively). *PVT1* also has two isoforms, *uc003ysl.2* and *uc010mdp.1*, and both of them are significantly overexpressed (adjusted *P*-values < 1E-10) in the cancer samples where *MYC* is also overexpressed.

The overexpression of *PVT1* lncRNA is induced by *MYC*, and is involved in the suppression of apoptosis (33,34). *PVT1* lncRNA and *MYC* protein expressions are correlated in primary human tumors, and the copy number of *PVT1* was increased in more than 98% of cancers that also showed an increase of *MYC* copies (35). Figure 4 shows the mRNA–lncRNA coexpression network centered on *PVT1* and *MYC*. The gene *MYC* has two isoforms, *uc003ysh.1* and *uc003ysi.2*, and both of them are significantly overexpressed (adjusted *P*-values 1.25E-14 and 4.56E-17, respectively) in kidney renal clear cell carcinoma Stage I (TPM = 1.60 and 77.47, respectively) with respect to the adjacent normal subset (TPM = 0.60 and 28.20, respectively). *PVT1* lncRNA also has two isoforms, *uc003ysl.2* and *uc010mdp.1*, and both of them are significantly overexpressed (adjusted *P*-values < 1E-10) in the cancer subset (TPM = 1.33 and 0.55, respectively) with respect to adjacent normal tissues (TPM = 0.03 and 0.03, respectively). As shown in Figure 4, there is a significant positive correlation between *MYC* isoform *uc003ysi.2* and PVT1 isoform *uc010mdp.1*.

## DISCUSSION

We processed massive data sets collected from NCBI GEO and TCGA in order to obtain complete transcriptome profiles, extract the differentially expressed protein-coding transcripts and lncRNAs involved in various cancer types and infer the lncRNA regulatory network. Although CRN was developed with the goal of collecting, processing, analyzing and visualizing all publically available cancer RNA-seq data, it is still limited by the characteristics of these data. Firstly, different subsets from the same RNA-Seq data set have different sizes, which can influence the power of statistical analysis. Secondly, the cancer RNA-Seq data were generated using different experiment designs, sequencing platforms, cDNA library protocols, genetic backgrounds (cell lines or patients) and so on. It is a great challenge to integrate such diverse data resources. We have therefore applied the strategy of only comparing subsets from the same data set. Thirdly, some clinical information has not yet been integrated into CRN yet, such as treatments, pharmaceutical therapies and survival data.

As more and more cancer RNA-Seq data sets accumulate in the TCGA and NCBI GEO databases, we will update our CRN database to ensure that it can empower biologists with comprehensive cancer transcriptome knowledge.
